# Serum amyloid A3 is required for normal weight and immunometabolic function in mice

**DOI:** 10.1371/journal.pone.0192352

**Published:** 2018-02-01

**Authors:** Jennifer L. Ather, Matthew E. Poynter

**Affiliations:** Vermont Lung Center, Division of Pulmonary Disease and Critical Care, Department of Medicine, University of Vermont, Burlington, VT, United States of America; Johns Hopkins University School of Medicine, UNITED STATES

## Abstract

Serum amyloid A (SAA) is an apolipoprotein that is robustly upregulated in numerous inflammatory diseases and has been implicated as a candidate pro-inflammatory mediator. However, studies comparing endogenous SAAs and recombinant forms of the acute phase protein have generated conflicting data on the function of SAA in immunity. We generated SAA3 knockout mice to evaluate the contribution of SAA3 to immune-mediated disease, and found that mice lacking SAA3 develop adult-onset obesity and metabolic dysfunction along with defects in innate immune development. Mice that lack SAA3 gain more weight, exhibit increased visceral adipose deposition, and develop hepatic steatosis compared to wild-type littermates. Leukocytes from the adipose tissue of SAA3-/- mice express a pro-inflammatory phenotype, and bone marrow derived dendritic cells from mice lacking SAA3 secrete increased levels of IL-1β, IL-6, IL-23, and TNFα in response to LPS compared to cells from wild-type mice. Finally, BMDC lacking SAA3 demonstrate an impaired endotoxin tolerance response and inhibited responses to retinoic acid. Our findings indicate that endogenous SAA3 modulates metabolic and immune homeostasis.

## Introduction

Serum amyloid A (SAA), an acute phase lipoprotein, has been studied for decades as a robust biomarker for a wide array of inflammatory and autoimmune disorders [1]. Multiple isoforms of this protein have been identified, including: SAA1 and 2, which are highly homologous and predominantly produced by the liver; SAA3, an acutely expressed isoform produced in non-primate mammals; and SAA4, which is constitutively expressed and does not increase in response to infection or injury [2]. In mice, SAA3 is the primary SAA isoform expressed in epithelial and hematopoietic cells [3], and we have previously demonstrated a rapid increase in *Saa3* gene expression in the lung in response to a variety of pro-inflammatory stimuli capable of inducing pulmonary innate and adaptive immune responses [4].

SAA has been reported to function as a circulating chaperone for retinoic acid (RA) [5]. RA is critical to normal metabolic homeostasis and healthy adipose development [6, 7], and has recently been found to promote adipose tissue browning via VEGF signaling [6]. SAA is also a potent opsonin of Gram-negative bacteria [8, 9] and can prevent viral entry into cells [10–12]. The revelation that SAA also performs chaperone functions for RA indicate that, in addition to being a biomarker and possibly contributing to inflammation, SAA may also have key homeostatic functions in lipid transport and inflammation resolution.

Dysfunctions in lipid metabolism are linked to obesity and metabolic disease. Obesity can lead to a variety of inflammatory complications, including hepatic steatosis [13], hypertension [14], and diabetes [15]. The increased storage of excess lipid in adipose tissue associated with obesity general results in increased storage of excess lipid and increased numbers of highly pro-inflammatory (M1) macrophages that cluster in “crown-like” structures and secrete elevated levels of IL-1β, IL-6, TNFα, and MCP-1 [16–18]. These M1 macrophages not only exacerbate the inflammatory environment, but also inhibit the anti-inflammatory (M2) functions of homeostatic adipose tissue macrophages, which include secreting IL-10, driving immune tolerance in other cell types, and clearing up apoptotic cells and debris [18–20].

Our objective was to determine the role of SAA3 in innate immune processes. We have generated a mouse lacking SAA3 that, instead of showing decreased pro-inflammatory responses, exhibits obesity and metabolic dysfunction, as well as increased pro-inflammatory myeloid cell profiles within the adipose tissue. Additionally, lack of SAA3 leads to altered bone marrow-derived dendritic cell (BMDC) cytokine secretion and repressed tolerogenic capabilities. These results indicate that SAA3 is critical to the development of the innate immune system as well as metabolic homeostasis.

## Materials and methods

### Mice

All animal experiments were conducted in accordance with the recommendations in the Guide for the Care and Use of Laboratory Animals of the National Institutes of Health, and efforts were made to minimize suffering. The work was approved by the University of Vermont’s Institutional Animal Care and Use Committee (protocol #12–018). Sodium pentobarbital was administered via intraperitoneal injection for euthanasia. C57BL/6 mice were purchased from Jackson Laboratories (Bar Harbor, ME). Targeted embryonic stem cells from C57BL/6N mice in which the protein coding sequence of *Saa3* was deleted were recovered from cryopreservation and injected into albino C57BL/6 embryos through the Knock Out Mouse Project (KOMP) at the University of California, Davis. Detailed information on the generation of the SAA3-deficient embryonic stem cells is available at: http://www.velocigene.com/komp/detail/13807. SAA3+/- mice were shipped to UVM and subsequently bred with C57BL/6J mice for 10 generations. All animals were maintained on 12 hour light/dark cycle and provided chow (~30% kCal from fat; TestDiet, St. Louis, MO) and water ad libitum in an AALAC-accredited facility. In some studies, mice were provided Low-fat (10% kCal from fat) and High-fat (60% kCal from fat) diets from Research Diets (New Brunswick, NJ) *ad libitum*. Mice were between 18 and 24 weeks of age for all studies except for the BMDC generation experiments, in which femurs from 8–10 week old mice were used.

### RNA isolation and quantitative PCR

Tissues were snap-frozen and pulverized in a mortar and pestle in liquid nitrogen. RNA was isolated from lung, liver, spleen, and kidney using the PrepEase kit (Affymetrix, Cleveland, OH) according to manufacturer’s instructions. Visceral adipose tissue (VAT) and subcutaneous adipose tissue (SCAT) underwent phenol:chloroform extraction and RNA was further purified using the PrepEase kit. cDNA was generated from total RNA using the iScript cDNA synthesis kit (Bio-Rad, Hercules, CA) according to manufacturer’s instructions. Q-PCR was performed using primer pairs (see S1 Table) and SYBR Green Universal Taq Mastermix (Bio-Rad). The levels of genes of interest were normalized to the house-keeping gene glyceraldehyde 3-phosphatase dehydrogenase (*Gapdh*), which did not differ between samples, and relative gene expression was calculated using the ΔΔC_T_ method as previously described [21].

### Generation of stromal vascular fraction (SVF) from visceral adipose tissue

Mouse VAT was collected and placed into digestion buffer (2 mg/ml collagenase, 100 mM HEPES, 120 mM NaCl, 50 mM glucose, 1 mM CaCl_2_, and 1.5% BSA) and processed using a GentleMacs dissociator (Miltenyi, Auburn, CA) according to manufacturer’s instructions. Following centrifugation at (100 x *g* and 500 x *g*), floating adipocytes and the SVF pellet were collected and processed for the preparation of cDNA.

### Immunohistochemistry

Liver tissues were fixed in 10% neutral buffered formalin, embedded in paraffin and 5 μm sections were cut and mounted on slides prior to staining with hematoxylin and eosin or Oil Red O. Hand processing, paraffin embedding, and staining were performed by the University of Vermont Medical Center Surgical Pathology Department. Hand processing, paraffin embedding, and staining were performed by the University of Vermont Medical Center Surgical Pathology unit. Stained tissue was imaged using an EVOS XL microscope (Life Technologies) at 20X (H&E) and 40X (Oil Red O).

### BMDC generation and stimulation

Femurs and tibiae from mice were flushed and marrow was plated at 1 x 10^6^ nucleated cells/ml. Cells were cultured for 6 days in RPMI-1640 supplemented with 10% FBS and 5% conditioned media from X63-GMCSF myeloma cells transfected with murine GM-CSF cDNA (kindly provided by Dr. Brent Berwin, Dartmouth College). Media was replaced on days 2 and 4, and the adherent and lightly adherent BMDC, predominantly CD11b^+^CD11c^+^ by flow cytometry, were collected on day 6. In some studies, BMDC were treated with 0.1 μM all-trans-retinoic acid (ATRA) (Sigma-Aldrich, St. Louis, MO), which was provided at the same time points at GM-CSF. In the tolerization studies, BMDC were treated with increasing doses of LPS (Invivogen, San Diego, CA) for 24 hours. Following this initial stimulation, cells were washed and DPBS, then challenged again with their tolerizing dose for a further 24 hours.

### Cytokine analysis

Cell supernatants were analyzed for protein secretion with the following assays: IL-1β and TNFα were measured by ELISA kits from BD Biosciences (San Jose, CA). IL-6, IL-10, and IL-23 were analyzed by ELISA kits from R&D Systems (Minneapolis, MN). SAA3 was measured either by ELISA or Milliplex assay (Millipore, Billerica, MA) according to manufacturer’s instructions. Serum cytokines (IL-1β, IL-6, IL-10, IL-23, and TNFα) were measured by a custom a Magnetic Luminex Assay (R&D Systems). Kit information, including limits of detection, can be found in S2 Table.

### Statistics

Data were analyzed by two-tailed unpaired t-test or one-way or two-way ANOVA and Bonferroni post-hoc test using GraphPad Prism 7 for Windows (GraphPad Software, Inc., La Jolla, CA.). A p value smaller than 0.05 was considered statistically significant.

## Results

### SAA3 deficiency promotes adult onset weight gain and dyslipidemia

**Fig 1:**
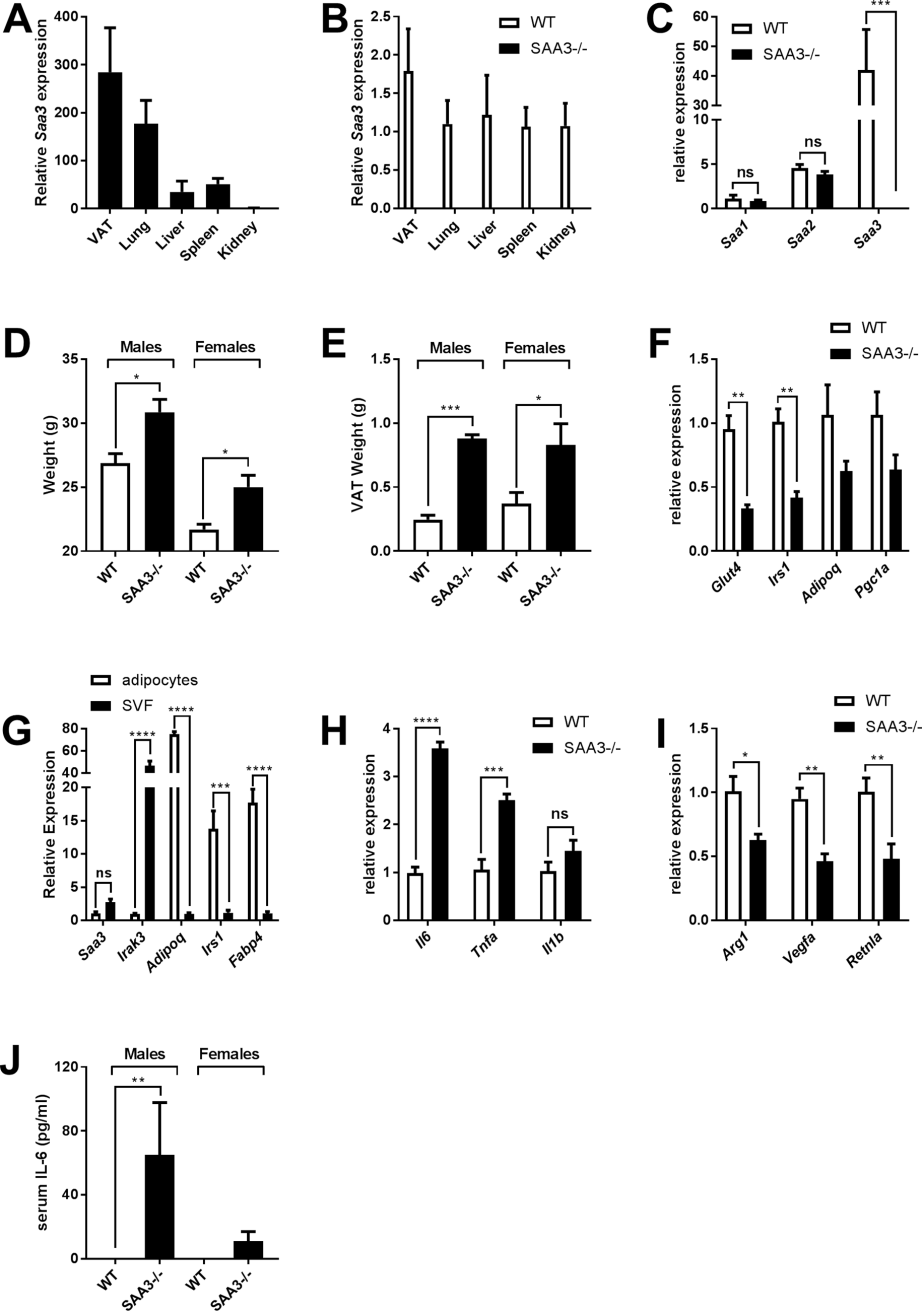
Characterization of SAA3-/- mice. Levels of basal *Saa3* expression from wild-type (WT) mice were measured from VAT, lung, liver, spleen, and kidney. Relative levels were compared to the kidney (A). WT and SAA3 knockout (SAA3-/-) littermates were analyzed by Q-PCR for *Saa3* gene expression in the same organs to confirm the deletion of *Saa3* in the SAA3-/- mice (B). Characterization of different SAA isoforms was performed by Q-PCR from VAT (C). WT and SAA3-/- mice were analyzed at 18 weeks of age for total body weight (D) and VAT weight (E). Insulin sensitive gene expression was analyzed by Q-PCR from VAT (F). Adipocyte and stromal vascular fractions were confirmed for cell-specific genes in wild-type mice (G). SVF from the VAT of WT and SAA3-/- mice were analyzed by Q-PCR for pro-inflammatory genes (H) and M2-related genes (I). Serum from WT and SAA3-/- mice was analyzed for circulating cytokines (J) n = 3-9/group. * = p<0.05, ** = p<0.01, *** = p<0.005, **** = p<0.001.

A comparative analysis of organs from wild-type (WT) mice indicated that, as has been previously published [22], the expression of *Saa3* is highest in the visceral adipose tissue (VAT) and the lung, with substantially less being expressed in the liver, spleen, and kidney (Fig 1A). We confirmed the effective elimination of *Saa3* expression in our knockout (SAA3-/-) littermates in the same tissues (Fig 1B), and then determined that levels of *Saa1* and *Saa2* were normal in the VAT of SAA3-/- mice (Fig 1C). In the course of utilizing mice for *in vivo* studies, we observed that SAA3-/- mice were consistently and significantly heavier than their wild-type littermates (Fig 1D). This weight gain appeared to be the result of increased adipose tissue mass in both male and female mice, as was observed for the VAT (Fig 1E). As there is a well-documented link between obesity and altered inflammatory responses [18, 23], we felt it prudent to explore the obese phenotype in these mice. Analysis of VAT from SAA3-/- mice revealed significant decreases in the expression of the insulin sensitive genes glucose transporter 4 (*Glut4*) and insulin receptor substrate-1 (*Irs1)* (Fig 1F). Increased mass of white adipose tissue in obesity is associated with elevated levels of tissue-resident pro-inflammatory macrophages. Separation of the adipocytes and stromal vascular fraction (SVF) in wild-type mice revealed no significant difference in the expression of *Saa3* in both cell populations, but otherwise a clear distinction between myeloid-specific genes (*Irak3)*, and adipocyte-specific genes (*Adipoq*, *Irs1*, and *Fabp4*) (Fig 1G). Analysis of the SVF from the VAT of WT and SAA3-/- littermates for the pro-inflammatory-M1-related genes interleukin-6 (*Il6*), interleukin-1beta (*Il1b*), and tumor necrosis factor alpha (*Tnfa*) revealed increased *Il6* and *Tnfa* expression in the SAA3-/- myeloid cells (Fig 1H). Furthermore, these cells had a downregulated expression of the anti-inflammatory, M2-related genes arginase-1 *(Arg1)*, vascular endothelial growth factor-alpha *(Vegfa)*, and resistin-like alpha (*Retnla*), encoding the protein known as Fizz1 (Fig 1I). The shift from an M2 to an M1 pro-inflammatory phenotype is consistent with those observed in obese adipose tissue [23, 24]. Serum from wild type and SAA3-/- mice was analyzed for cytokine content. IL-1β, IL-10, IL-23, and TNFα were below the limits of detection of the assay, however both male and female SAA3-/- mice demonstrated variable levels of IL-6, which in males was significantly increased in the SAA3-/- mice compared to WT (Fig 1J).

### The obese state in SAA3-/- mice is accompanied by early indicators of non-alcoholic fatty liver disease

**Fig 2:**
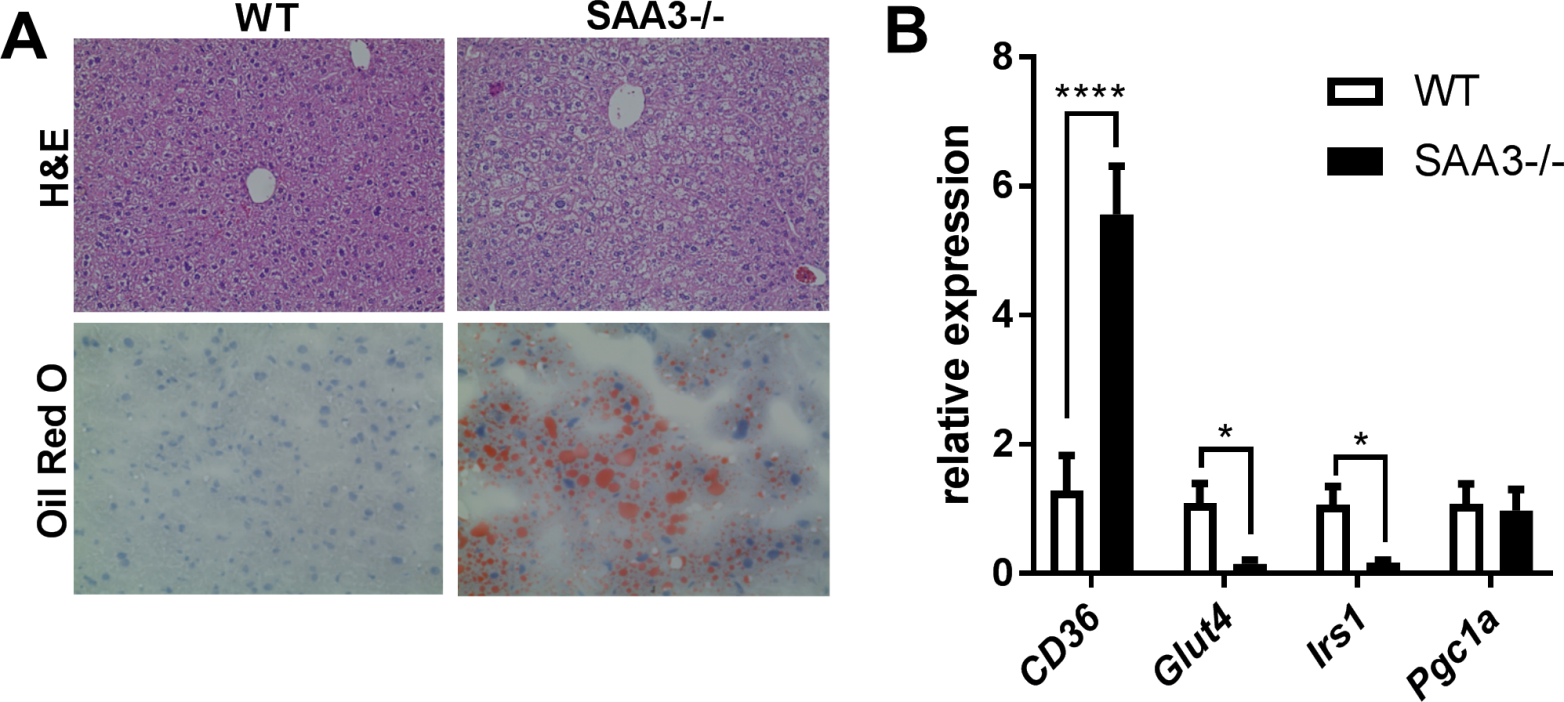
Livers from SAA3-/- mice demonstrate early signs of non-alcoholic fatty liver disease. Liver tissue from WT and SAA3-/- mice at 18 weeks of age were fixed in 10% neutral-buffered formalin and stained for H&E (A, top row) and Oil-Red O (A, bottom row). Representative images are presented. Q-PCR analysis from liver was performed for genes that regulate insulin sensitivity (B). n = 3-4/group. *p = <0.05, **** = p<0.001.

Liver tissue from the obese SAA3-/- mice was visibly different from that of WT littermate controls. Histological analysis revealed increased vacuolation in SAA3-/- hepatocytes (Fig 2A, top row) and staining with Oil Red O confirmed increased lipid content in the SAA3-/- hepatic cells compared to WT tissue (Fig 2A, bottom row). SAA3-/- mice demonstrated increased gene expression for *CD36*, a fatty acid transporter linked to obesity and metabolic disease, and decreased expression of the insulin-sensitive genes *Glut4* and *Irs1*, with no change in the expression of peroxisome proliferator-activated receptor γ coactivator-1 alpha (*Pgc1a)*.

### Expression of insulin-sensitive genes are decreased in SAA3-/- mice fed a high-fat diet for 1 week

**Fig 3:**
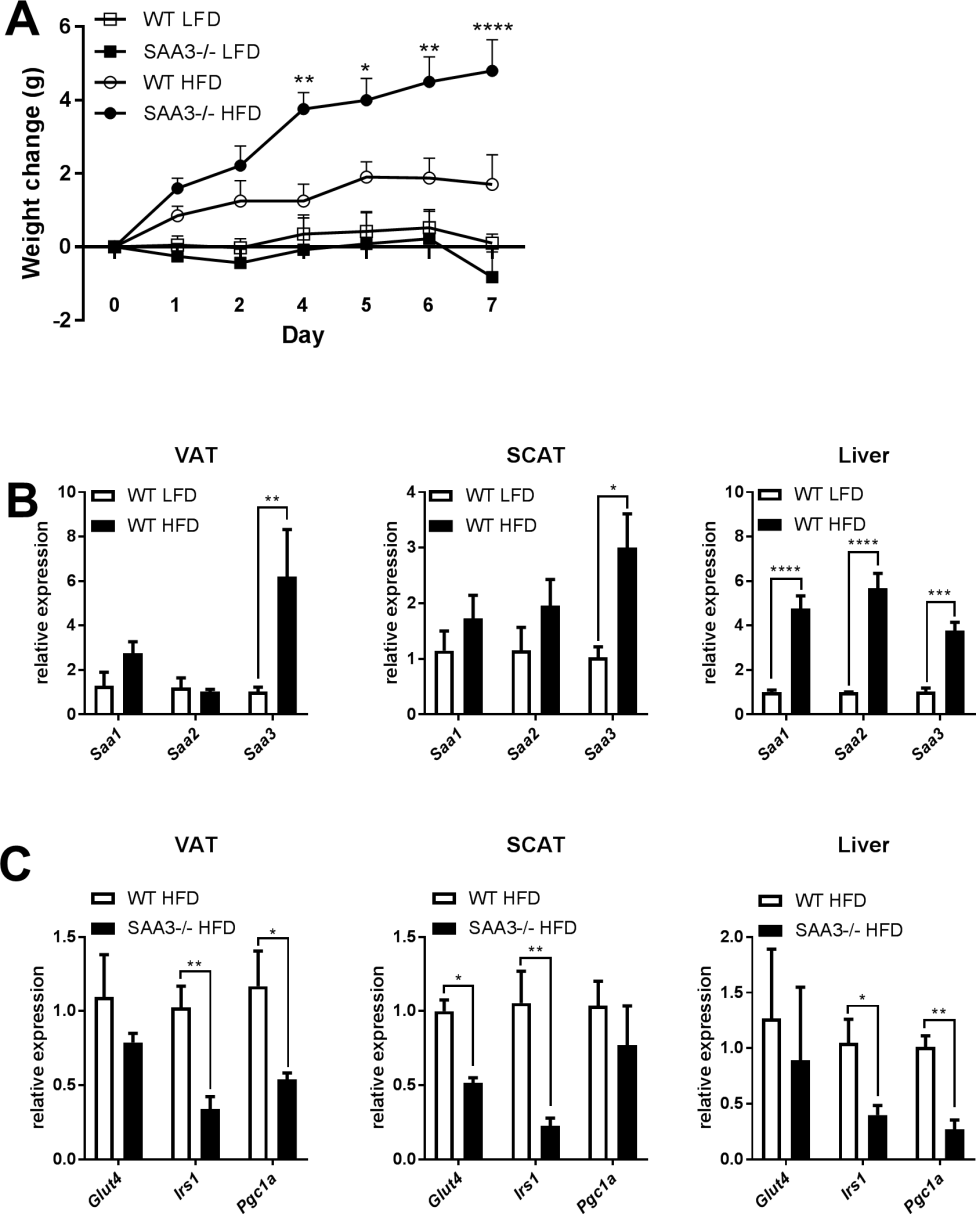
High fat diet leads to more substantial weight gain and insulin-sensitive gene repression in SAA3-/- mice. 18 week old wild type (WT) and SAA3-/- mice were placed on high-fat diet (HFD = 60% kCal from fat) or low-fat diet (LFD = 10% kCal from fat) for 7 days. Total body weight was measured each day (A). Comparisons displayed are between HFD-fed WT and SAA3-/- mice. The expression of SAA isoforms in VAT was analyzed from WT mice on the LFD and HFD (B). Insulin-sensitive gene expression was analyzed by Q-PCR from VAT, subcutaneous adipose tissue (SCAT), or liver of WT and SAA3-/- mice fed HFD (C). n = 4-5/group. * = p<0.05, ** = p<0.01, *** = p<0.005, **** = p<0.001.

The inhibition of insulin-sensitive gene expression in the VAT and liver of the SAA3-/- mice, along with their increased weight gain, suggested they may exhibit a predisposition for exacerbated responses to high-fat diet. Wild-type and SAA3-/- littermate mice were fed either a high-fat diet (HFD) with 60% kCal from fat (lard) or a 10% kCal from fat low-fat diet (LFD) for one week. Previous studies have demonstrated that acute exposure to high-fat diet feeding can induce rapid, systemic changes in hormone signaling and gene alteration [25, 26]. Even at this early time point, all mice fed HFD gained significantly more weight compared to those on the LFD, with the SAA-/- mice fed HFD increasing their weight significantly compared to WT mice fed HFD (Fig 3A). Feeding the HFD led to significant increases in VAT, liver, and subcutaneous adipose tissue (SCAT) *Saa3* gene expression in the WT mice, compared to WT mice fed LFD. Gene expression levels of *Saa1*and *Saa2* were unaffected in both adipose tissue types; however, they, along with *Saa3*, were increased in the liver by feeding the HFD. (Fig 3B). SAA1 and SAA2 production from the liver is critical to the acute phase response, and though their expression is not altered basally in the SAA3-/- mice, the alterations observed in response to HFD indicate a potential for a differential acute phase response in mice lacking SAA3 in response to stimuli. SAA3-/- mice that were fed HFD demonstrated significant decreases in *Irs1* and *Pgc1a* in VAT and liver, and in *Glut4* and *Irs1* in SCAT compared to WT mice fed HFD (Fig 3C).

### Lack of SAA3 exacerbates the dendritic cell response to LPS and leads to dysfunctional endotoxin tolerance

**Fig 4:**
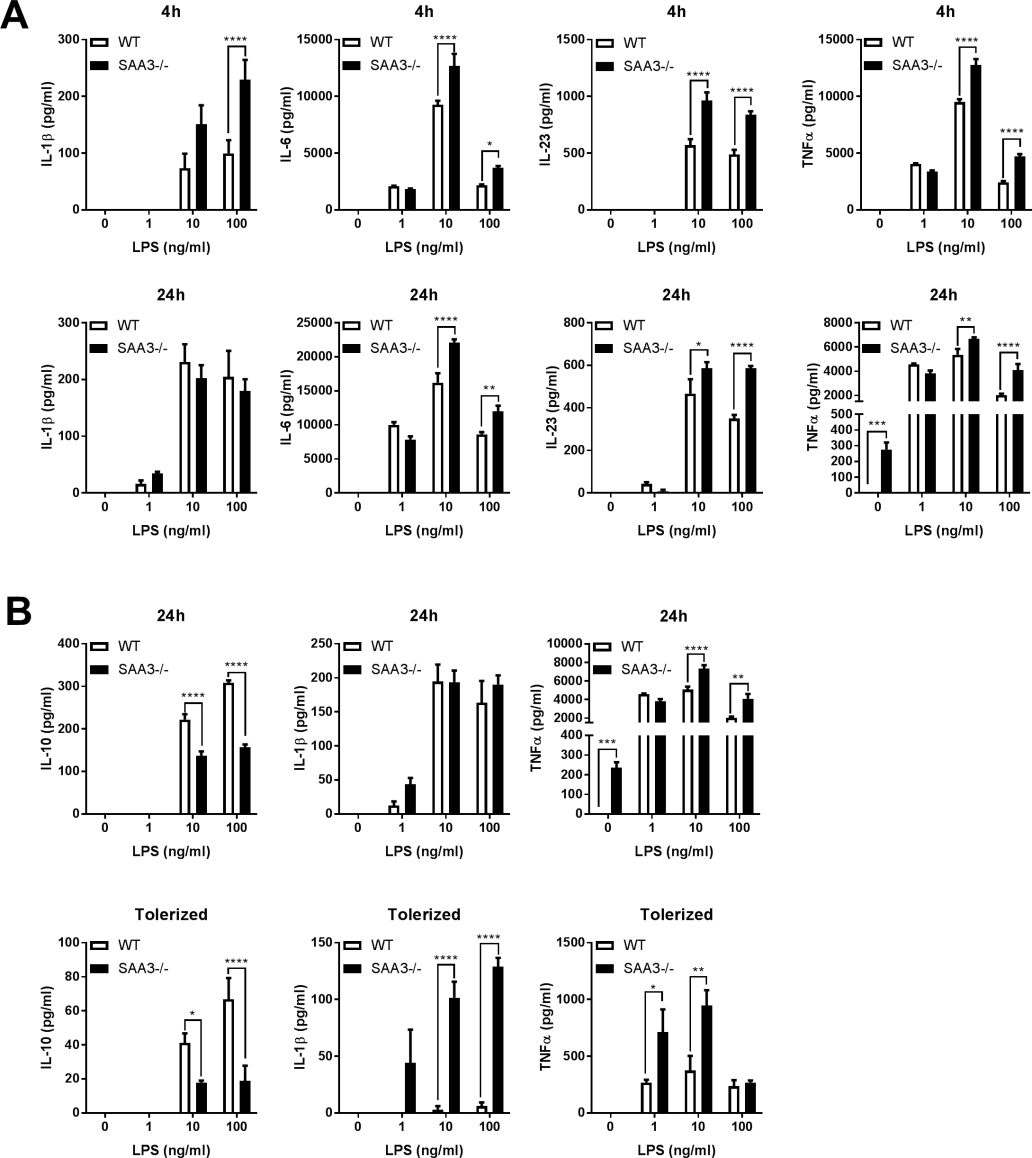
SAA3-/- mice display altered myeloid cell responses. Bone marrow derived dendritic cells generated from WT and SAA3-/- mice were challenged with increasing doses of LPS for 4 and 24 hours. IL-1β, IL-6, IL-23, and TNFα were measured from cell supernatants by ELISA (A). BMDC were challenged for 24 hours with increasing doses of LPS (24h). These cells were then restimulated with the same dose of LPS for a further 24 hours (Tolerized) and supernatants were analyzed for IL-1β, IL-10, and TNFα by ELISA (B). n = 3-7/group. * = p<0.05, ** = p<0.01, *** = p<0.005, **** = p<0.001.

We next analyzed bone marrow progenitors that were differentiated *in vitro* into myeloid dendritic cells (BMDC), a cell type modeling those in vivo that participate in innate immune responses and that function as antigen presenting cells to initiate adaptive immune responses [27]. The BMDC were challenged with increasing doses of LPS, a component of gram-negative bacterium, and cytokines from cell-free supernatants were measured at 4 and 24 hours. At 4 hours, BMDC from SAA3-/- mice produced significantly increased levels of IL-1β, IL-6, IL-23, and TNFα in response to 10 or 100 ng/ml LPS compared to BMDC from wild-type mice (Fig 4A, top row). Increased secretion of IL-6, IL-23, and TNFα remained at 24 hours (Fig 4A, bottom row). It is also of note that by 24 hours, untreated SAA3-/- BMDC had secreted ~300 ng/ml of TNFα even in the absence of LPS treatment, whereas BMDC from unstimulated WT mice had undetectable TNFα levels. Spontaneous secretion of pro-inflammatory cytokines and exacerbated responses to LPS are suggestive of dysregulated inflammatory resolution. Previous studies indicate that after initial exposure to LPS, a secondary restimulation with LPS will result in decreased secretion of pro-inflammatory cytokines such as IL-1β and TNFα, and a compensatory increase in the anti-inflammatory cytokine interleukin-10 (IL-10), an effect known as “endotoxin tolerance” [28, 29]. In a separate experiment, BMDC from WT and SAA3-/- mice were challenged with LPS for 24 hours (Fig 4B, top row), and subsequently rechallenged with LPS for another 24 hours (Fig 4B, “Tolerized” bottom row). In the initial response to LPS, SAA3-/- BMDC produced less IL-10 and more TNFα than WT controls (Fig 4A, top row). After restimulation, WT BMDC continued to secrete IL-10, but the production of both IL-1β and TNFα were significantly decreased (Fig 4B, bottom row, white bars). However, SAA3-/- BMDC continued to strongly secrete IL-1β and TNFα, and maintained lower levels of IL-10 (Fig 4B, bottom row, black bars), indicating weakened endotoxin tolerance in these cells.

### SAA3 is required for normal BMDC responses to retinoic acid

**Fig 5:**
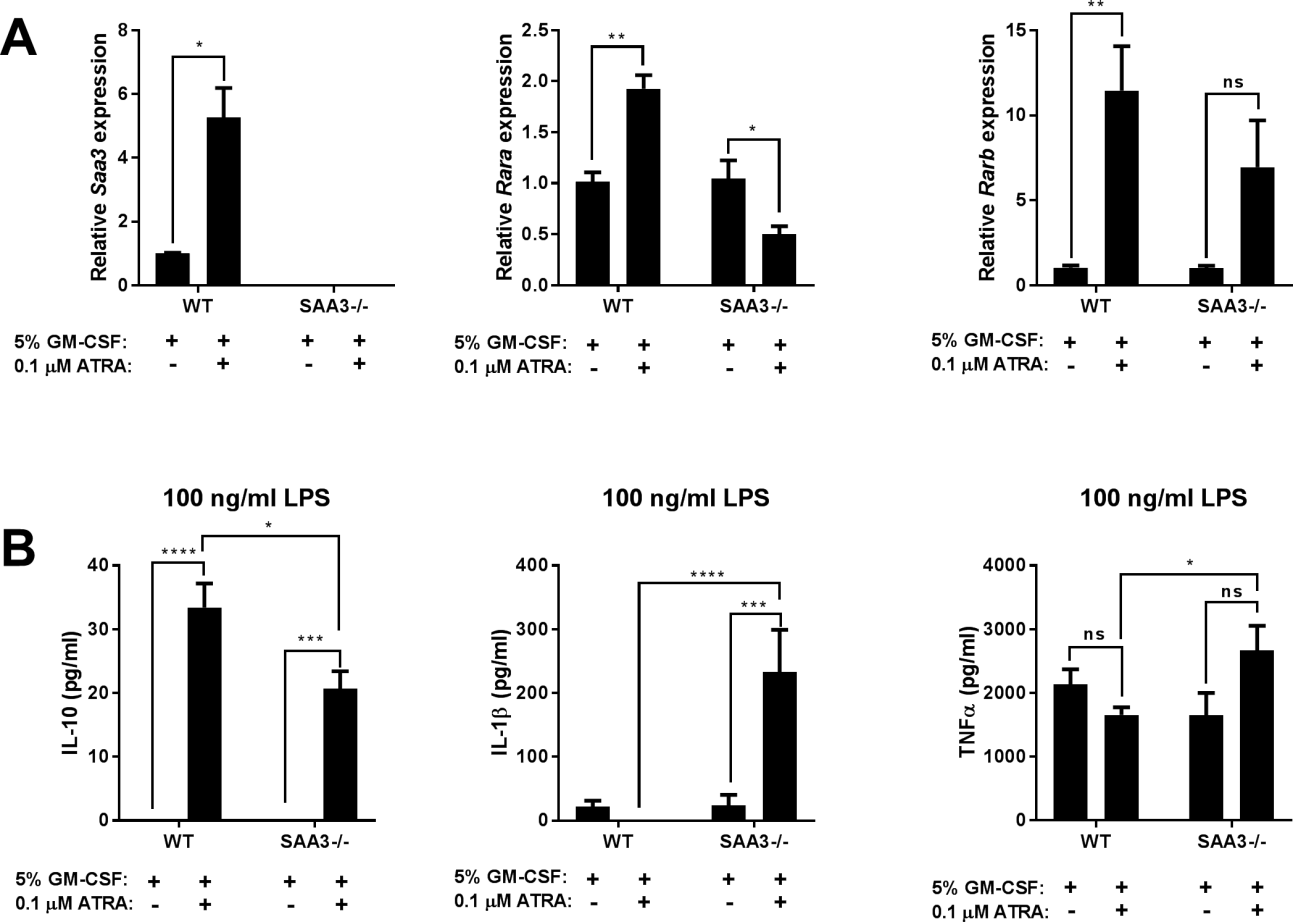
Lack of SAA3 inhibits dendritic cell responses to retinoic acid. Bone marrow derived cells were isolated from WT and SAA3-/- mice and differentiated into dendritic cells for 7 days in the presence of 5% GM-CSF or 5% GM-CSF + 0.1 μM all-trans-retinoic acid (ATRA). At day 7, cells were analyzed for *Saa3*, *Rara*, and *Rarb* gene expression by Q-PCR (A). At day 7, cells were also challenged with 100 ng/ml LPS for 24 hours, and IL-10, IL-1β, and TNFα were measured from cell supernatants by ELISA (B). n = 3-7/group. * = p<0.05, ** = p<0.01, *** = p<0.005, **** = p<0.001.

Retinoic acid has the ability to induce tolerogenic myeloid cells [30, 31]. BMDC were generated as in the previous experiment, with the addition of 0.1 μM all-trans-retinoic acid (ATRA) provided with each feeding of GM-CSF over a 7 day differentiation period. WT cells differentiated in the presence of ATRA demonstrated an increased expression of *Saa3*, as well as increased expression of both retinoic acid receptors alpha (*Rara*) and beta (*Rarb*) (Fig 5A). The SAA3-/- BMDC failed to significantly increase expression of *Rara*. BMDC at day 7 were also washed, plated, and challenged with 100 ng/ml LPS for 24 hours. Differentiation with ATRA allowed for the LPS-induced release of IL-10 from WT cells, which was diminished from the SAA3-/- cells. Strikingly, while ATRA inhibited IL-1β release from WT cells, it significantly increased the production of this cytokine from SAA3-/- cells (Fig 5B).

## Discussion

Serum amyloid A has been observed to increase ~1000 fold in the serum in response to a number of infection and injury models [1]. Additionally, tissue-specific expression of *Saa3* increases in mice in response to a variety of stimuli [4]. Given its rapid and robust increase under inflammatory and autoimmune conditions, it has long been speculated that SAA is a mediator of the inflammatory process. We and others have published data suggesting this is the case using a commercially available recombinant form of SAA [4, 32, 33]. However, the *E*. *coli*-derived recombinant form of SAA that is almost uniformly used by investigators elicits strong pro-inflammatory responses not shared with the endogenous form of SAA [34]. Specifically, whereas recombinant apoSAA promoted neutrophil activation and pro-inflammatory cytokine production, human plasma containing highly elevated levels of SAA displayed neither of those effects. These results imply that further characterization of the differences in pro-inflammatory activities between recombinant and endogenous SAA is warranted. Consequently, our observations suggest that the robust production of SAA3 in response to injury or infection may be an attempt to maintain homeostatic function; that is, that endogenously produced SAA may exert a protective or pro-resolving effect in the immune response.

Consistent with this notion, our results indicate that mice lacking SAA3 develop increased adipose deposition, metabolic dysfunction, and exacerbated pro-inflammatory responses from innate immune cells. While conducting our studies, another group generated SAA3-/- mice in the same manner as we did and reported that the SAA3-/- mice fed a high-fat, high-sucrose, high-cholesterol diet gained less weight and had less adipose infiltration with macrophages [35]. This group also reported decreases in the expression of *Saa1* and *Saa2* in the livers of SAA3-/- mice, similar to what we observed after one week of HFD, which they speculated may link SAA3 functionally to the acute phase response and to integrated immune response changes in general. Reasons for the differences in weight gain by SAA3-/- mice between this study and our own are unclear, although the dissimilarities of dietary sucrose and cholesterol content in the high-fat diets could be part of the explanation. SAA1 and SAA2 are found in the circulation bound to HDL, and a link between these forms of SAA and metabolic diseases has been speculated upon [36, 37]. However, SAA3 does not bind HDL to the same extent as SAA1 [38, 39], and is found at far lower concentrations in the serum, indicating that acute production of SAA3 is more critical in localized tissue responses. How SAA3 specifically affects metabolic homeostasis and regulates adipokine and insulin-sensitive gene expression is less clear. It has recently been reported that SAA acts as a chaperone for retinoic acid (RA), a metabolite that is critical to metabolic homeostasis [40]. Although we found no difference between WT and SAA3-/- mice in the basal level of retinoic acid receptor-alpha gene expression in VAT or liver tissue homogenates, we did observe that dendritic cells from mice lacking SAA3 failed to upregulate the expression of this receptor directly in response to retinoic acid treatment. It is tempting to speculate that SAA3 may chaperone RA or other critical metabolites, leading to metabolic dysfunction in mice that lack this specific isoform of SAA. This may also account for the altered innate immune cell profiles in the SAA3-/- mice.

Questions remain regarding the intersection between the metabolic dysfunction observed in mice lacking SAA3 and the alterations displayed by their immune cells. Recent reviews have highlighted the connection between metabolism and immunity [41, 42], and call for a more integrative view of the two fields. Our data demonstrating a failure to acquire endotoxin tolerance in BMDCs that lack SAA3 is intriguing in light of the finding that feeding a high fat diet leads to systemic metabolic endotoxemia that worsens obesity and exacerbates inflammatory responses in mice [43]. Metabolic endotoxemia has also been observed in obese human patients, in which it correlates to a severe decrease in endotoxin-specific IgM, indicating a loss of endotoxin neutralization and tolerance due to chronic LPS exposure from altered obese gut microbial species [44]. These studies indicate that increased levels of endotoxin exposure, from Western diet-induced microbiota outgrowth, may alter the tolerance threshold, leading to systemic inflammation, decreased glucose tolerance, and poor responses to infection. Expression of SAA3 in the gut is regulated by the microbiome [45], and SAA3 can bind directly to bacteria, leading to the possibility that SAA3 functions to regulate homeostatic, tolerogenic signaling of endotoxin produced by commensal microbiota. Further investigation is also required to determine whether the generation of immunometabolic imbalance observed in the adipose-resident leukocytes in SAA3-/- mice is due to the absence of SAA3 in the bone marrow during hematopoiesis, or an effect exerted systemically by the secretion of pro-inflammatory mediators from the adipocytes themselves.

The results presented herein demonstrate that mice lacking SAA3 have a predisposition for obesity and metabolic syndrome, and exhibit altered immune cell development. Inflammatory responses are worsened in SAA3-/- cells in response to LPS, and endotoxin tolerance is defective. Ultimately, these data highlight the critical role of metabolic dysfunction in impaired immune responses, and implicates SAA as a key mediator at the crossroads of metabolism and immunity.

## Supporting information

S1 Table Q-PCR primer sequences. Forward and reverse primer sequences for the mouse genes analyzed by Q-PCR.
(TIF)

S2 Table Product information. Product purchasing information for reagents, kits, and special diets used in this manuscript. Limits of detection are provided for ELISA and multiplex assays.
(TIF)
