# Supplementary figures and images for: Serum amyloid A3 is required for normal weight and immunometabolic function in mice
Source: PLoS One. 2018 Feb 1;13(2):e0192352. doi: 10.1371/journal.pone.0192352 (PMC5794179; doi:10.1371/journal.pone.0192352)

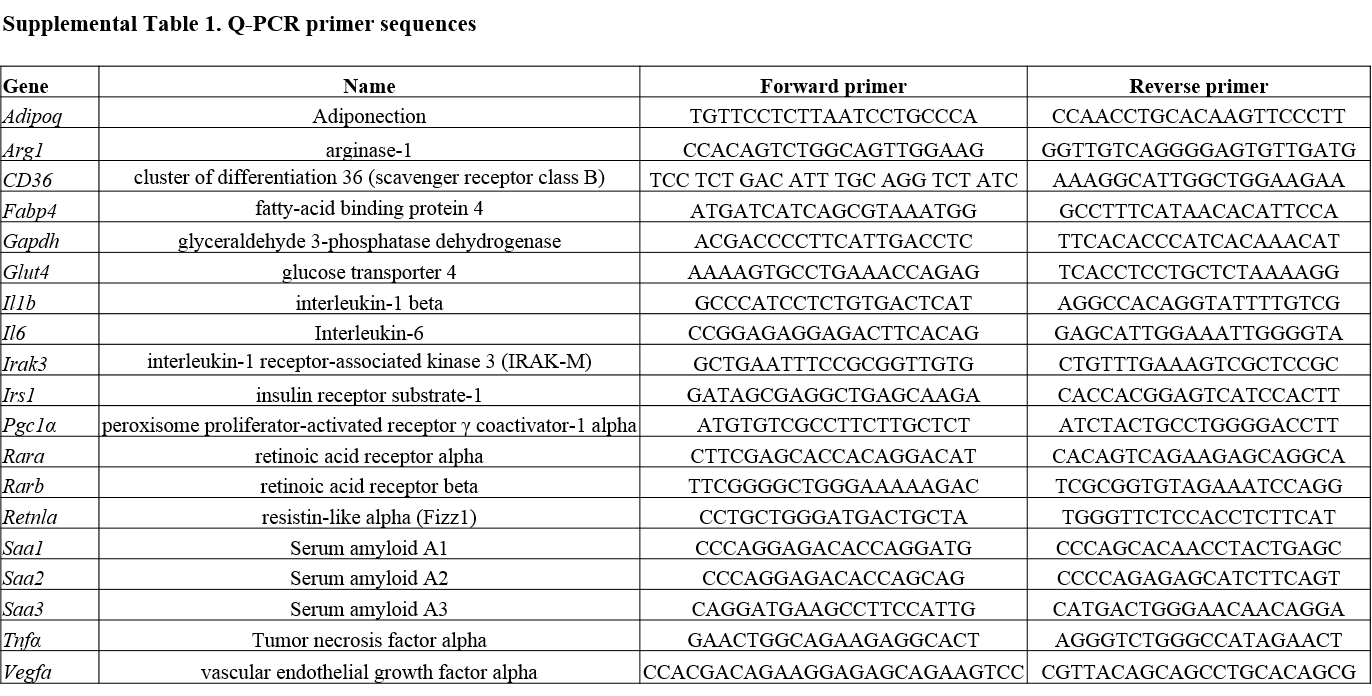

Supplement: S1 Table — Forward and reverse primer sequences for the mouse genes analyzed by Q-PCR. (TIF) [file pone.0192352.s001.tif]

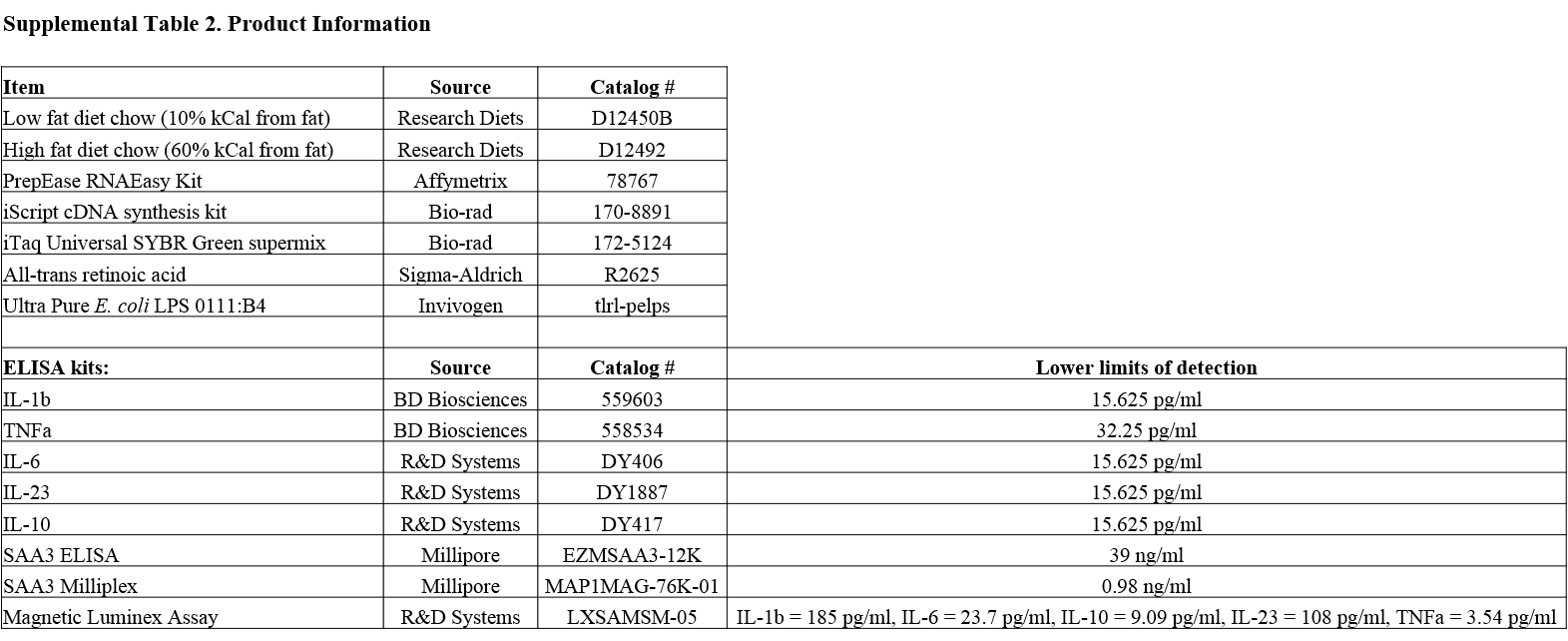

Supplement: S2 Table — Product purchasing information for reagents, kits, and special diets used in this manuscript. Limits of detection are provided for ELISA and multiplex assays. (TIF) [file pone.0192352.s002.tif]
